# Photo-Fries-based photosensitive polymeric interlayers for patterned organic devices

**DOI:** 10.1007/s00339-012-6853-2

**Published:** 2012-03-22

**Authors:** Alberto Montaigne Ramil, Gerardo Hernandez-Sosa, Thomas Griesser, Clemens Simbrunner, Thomas Höfler, Gregor Trimmel, Wolfgang Kern, Quan Shen, Christian Teichert, Günther Schwabegger, Helmut Sitter, Niyazi Serdar Sariciftci

**Affiliations:** 1 Linz Institute for Organic Solar Cells (LIOS), Institute of Physical Chemistry, Johannes Kepler University, Altenbergerstrasse 69, Linz, 4040 Austria; 2 Institute of Semiconductor and Solid State Physics, Johannes Kepler University, Altenbergerstrasse 69, Linz, 4040 Austria; 3 Institute of Chemistry of Polymeric Materials, University of Leoben, Otto Glöckel-Strasse 2, 8700 Leoben, Austria; 4 Institute for Chemistry and Technology of Materials, Graz University of Technology, Stremayrgasse 9, Graz, 8010 Austria; 5 Institute for Physics, University of Leoben, Franz-Josef Strasse 18, 8700 Leoben, Austria

## Abstract

This work reports on the investigation of the photosensitive polymer poly(diphenyl bicyclo[2.2.1]hept-5-ene-2,3-dicarboxylate) (PPNB), which undergoes the photo-Fries rearrangement upon illumination with UV-light, used as interfacial layers in organic electronic devices. Two cases were investigated: the use of a blend of PPNB with poly-vinylcarbazole (PVK) as an interlayer in para-sexiphenyl (PSP) based organic light emitting diodes (OLEDs) and the use of PPNB as gate dielectric layer in organic field effect transistors (OFETs). The photo-Fries rearrangement reaction causes a change of the polymer chemical structure resulting in a change of its physical and chemical properties. The electroluminescence spectra and emission of the PSP OLEDs are not affected when fabricated with a non-UV-illuminated PPNB:PVK blend. However, the electroluminescence is totally quenched in those OLEDs fabricated with UV-illuminated PPNB:PVK blend. Although the dielectric constant of PPNB increases upon UV-treatment, it is demonstrated that those OFETs built with UV-treated PPNB as gate dielectric have lower performance than those OFETs built with non-UV-treated PPNB. Furthermore, the effect of the UV-illumination of PPNB and PPNB:PVK blend on the growth of the small molecules C_60_ and PSP has been studied by atomic force microscopy. Using photolithography, this kind of photochemistry can be performed to spatially control and tune the optical and electrical performance of organic electronic devices.

## Introduction

During the past years, electronic devices based on organic semiconductors attracted much interest within industrial and scientific research. Based on chemical synthesis, a broad variety of organic molecules are produced for applications like organic photovoltaics [1–3], organic light emitting diodes (OLEDs) [4–7], and organic field effect transistors (OFETs) [8, 9]. The possibility of cheap, fast, and easy device fabrication is highly attractive from a commercial and industrial perspective.

The production of electronic devices and their integration requires the patterning of several layers involved in their fabrication. Manufacturing and patterning processes which provide high throughput, reliability, and reproducibility are needed to fully exploit the large scale production possibilities. Several techniques have been tested for the structuring of organic materials, e.g., embossing, printing, and photolithographic patterning [10–14]. Among these methods, photolithographic techniques provide a versatile and powerful means of patterning. It is added as an advantage that the image of the mask features can be magnified or demagnified when projected onto the substrate and it also provides an excellent spatial resolution [15–17]. In addition, photolithography is a well-established technique, e.g., in the silicon semiconductor industry. However, most of the standard lithographic techniques use wet chemical etch processes and solvents.

In the recent past, new polymers which undergo a photo-Fries rearrangement upon UV illumination have been synthesized and their applications in optoelectronics have been studied. It has been shown the selective modulation of the refractive index, surface functionalization, and patterning of those photosensitive polymers upon UV illumination through shadow mask [18, 19]. It has been demonstrated that due to the photo-Fries-rearrangement the surface polarity of the polymer poly(diphenyl bicyclo[2.2.1]hept-5-ene-2,3-dicarboxylate) (PPNB) increases and, therefore, such treatment influence the growth of para-sexiphenyl (PSP) deposited on the polymer [20]. A similar influence on the growth behavior of pentacene was observed by using a photo-acid generator polymer. By small change of the surface polarity upon illumination, it was possible to control the grain size of pentacene, and thus the mobility of charge carriers in the OFETs [21].

In this contribution, we present the study realized on the use of the polymer PPNB, which undergoes a photo-Fries rearrangement upon irradiation with UV light, and its blend with poly-vinylcarbazole (PVK) as photosensitive layers in OFETs and OLEDs, respectively, built with evaporable small organic molecules as semiconducting active films. Furthermore, the indirect patterning of the evaporable semiconducting layer by means of the UV patterning of the underlying PPNB or PPNB:PVK film has been investigated.

**Fig. 1:**
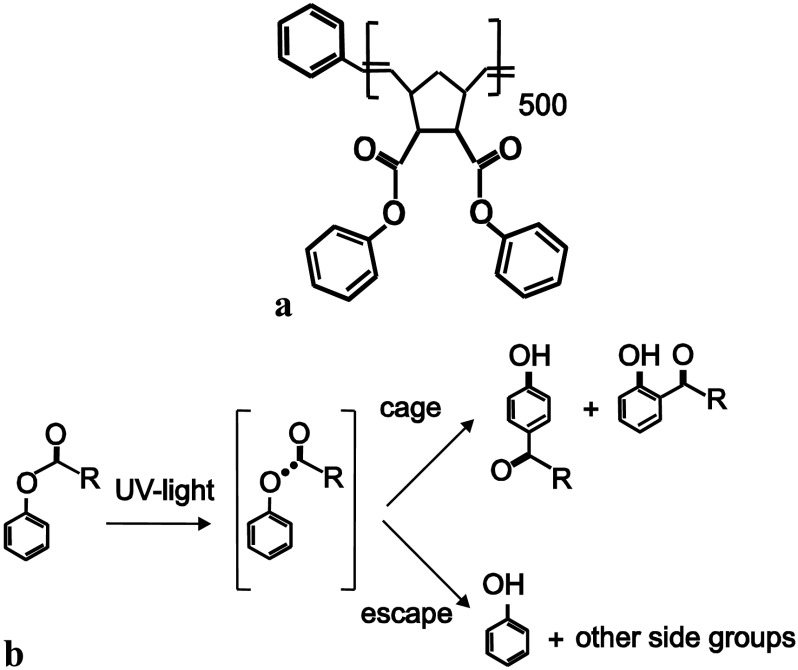
(**a**) Structure of the PPNB. (**b**) Scheme of the photo-Fries rearrangement which takes place in PPNB upon UV illumination

The structure of the PPNB polymer is shown in Fig. 1(a). The polymer PPNB undergoes a photo-Fries reaction upon irradiation with UV-light of wavelength shorter than 280 nm; see Fig. 1(b). Thereby, aryl esters groups are transformed to hydroxyketones resulting in an increase of the refractive index of the material [22]. Moreover, phenolic OH groups are generated in UV illuminated regions being responsible for a change of surface polarity as well. In that sense, it has been demonstrated that the growth of organic crystallites can be influenced in a controlled and accurate way by selective UV irradiation of the PPNB polymer by using shadow masks [20].

**Fig. 2:**
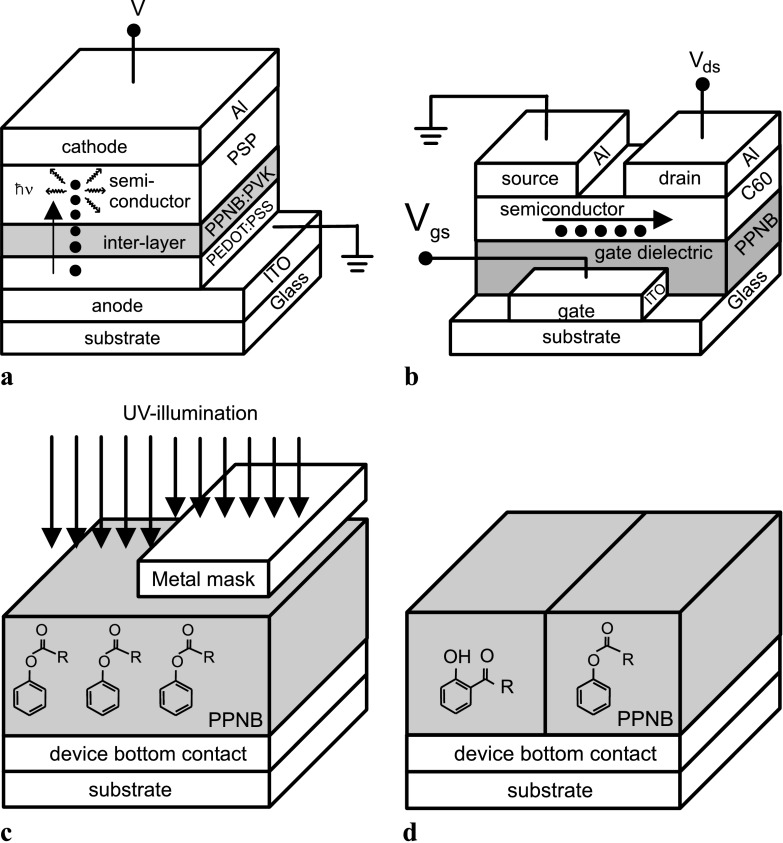
(**a**) and (**b**) are schemes of the OLED and the OFET, respectively, and the transport of electric charges within them. In these figures, the electric charges are represented by *black circles*. (**c**) Scheme of UV illumination of PPNB through shadow mask. The chemical compound correspond to the aryl-ester contained in the PPNB molecule. (**d**) Shows the aryl esters groups transformed to hydroxyketones in the half of the sample UV-illuminated

In OLEDs, the electrical current flows through the whole volume of the device [23]. The electrons and holes are injected from the electrodes into the semiconductor where they recombine emitting photons; see Fig. 2(a). In OFETs, the transport of charge is restricted just to the interface of the semiconductor to the gate dielectric [23–26]. By applying a voltage between gate and source contacts, the electric charges are injected in the semiconductor and accumulated at its interface to the gate dielectric (formation of the channel). The accumulated charges in the channel then moved from the source to the drain electrode under the potential difference; see Fig. 2(b).

We chose the small molecule organic semiconductors PSP and C_60_ as active materials in the presented OLEDs and OFETs, respectively. Both materials and their use in organic devices have been extensively investigated during the last years. PSP has been among the first organic materials studied for application in OLEDs [27]. It is well known as a blue light emitter, energy gap equal to 3.1 eV, with a high electroluminescence quantum yield and high chemical stability [28]. Furthermore, laser emission has been obtained from self-assembled PSP nanoneedles grown by Hot Wall Epitaxy on a crystalline substrate [29, 30]. C_60_, an n-type organic semiconductor with an energy gap of 1.8 eV has shown its high potential as an active layer in OFET [31–34]. As already reported in the literature, mobility values as high as 3 cm^2^ V^−1^ s^−1^ could be achieved for C_60_ based n-type OFETs with organic gate dielectrics [34, 35].

## Experimental

### Device fabrication

The PPNB synthesis is reported elsewhere [22].

For the fabrication of the OLEDs, the anode was prepared by spin coating poly[3,4-(ethylenedioxy) thiophene]: poly(styrene sulfonate) (PEDOT:PSS) on Indium Tin Oxide (ITO) covered glass. A 10 mg/mL solution of PPNB:PVK, (1:1 in mass), in chloroform was prepared and stirred for 12 h. It was spin cast on the anode resulting in a 80 nm thick layer of PPNB:PVK. The layer was UV illuminated during 20 minutes with UV light of *λ*=254 nm. During the UV-illumination part of the sample was covered with a metal mask. In that way, one part of the substrate was UV irradiated while the other half was kept in dark. Finally, a 15 nm thick layer of PSP was thermally evaporated by hot wall epitaxy during 45 minutes at a substrate temperature of 100 °C and a residual pressure of 9×10^−6^ mbar, followed by the evaporation of 50 nm of Al as complementary contact. Figure 2(a) shows the scheme of the fabricated OLED. The resulting sample contains both types of OLEDs fabricated on UV illuminated and non UV illuminated PPNB:PVK.

The n-type C_60_/PPNB transistors were fabricated in staggered configuration; see Fig. 2(b). PPNB is an insulator and, therefore, was used directly as gate dielectric. A solution of 40 mg of PPNB dissolved in 1 mL of chlorobenzene was drop casted on etched ITO laying on glass substrate which acts as the bottom gate contact. Afterward, the PPNB was dried in a vacuum oven at 40 °C for 1 hour. The resulting photo-sensitive PPNB dielectric film was 3.4 μm thick. A set of the obtained PPNB samples was kept in dark while another set was illuminated for 90 minutes with UV-light of 254 nm wavelength. The 100 nm thick C_60_ active layer was deposited on top of the PPNB dielectric films by thermal evaporation at a growth rate of 0.15 nm s^−1^ and a residual pressure of 5.0×10^−6^ mbar. The devices were finalized with the thermal evaporation, through shadow mask, of the 80 nm thick top drain and source Al contacts at 0.04 nm s^−1^ and a residual pressure of 6.0×10^−6^ mbar.

For the UV illumination of PPNB:PVK and PPNB films, the light was generated by an ozone free low pressure Hg lamp (Hereaus Noblelight). The light intensity (power density) at the sample surface was measured with a spectroradiometer (Solatell, Sola Scope 2000TM, measuring the range from 230 to 470 nm). All UV irradiations of the PPNB:PVK and PPNB polymer samples were conducted under inert gas atmosphere (nitrogen with a purity >99.95 %). The integrated power density of the UV light, *λ*=254 nm, impinging on the films was 1.4 mW cm^−2^. Figure 2(c) illustrates schematically the experimental setup we used where the deposited PPNB or PPNB:PVK films were UV illuminated prior to the deposition of the semiconductor layers. As shown in Fig. 2(d), upon UV illumination a photo-Fries reaction takes place in the PPNB, and consequently the aryl esters groups are transformed to hydroxyketones.

In order to optimize the UV-illumination time of the thick PPNB film used as gate dielectric in the OFET, its photoreaction was monitored by observing the absorption caused by the ketone at a wavenumber of 1630 cm^−1^ as function of irradiation time. For that purpose, following the procedures used for the OFETs, a PPNB film was deposited on a calcium fluoride (CaF_2_) substrate and illuminated with UV light. The experiment shows a saturation of the ketone absorption after approximately 80 minutes of irradiation. Therefore, the PPNB films used in the OFETs were illuminated during 90 minutes in order to assure the highest amount of film conversion due to UV-illumination.

The ITO contact, PPNB layer, top Al contact and UV-illumination of the metal-insulator-metal (MIM) structures were done following the same procedures as those of the OFET described above.

### Layer and device characterization

For the electrical characterization, the OLEDs were placed inside a sample box filled with nitrogen. A chip carrier holds the sample and electrodes were soldered with indium to it. In order to measure electroluminescence (EL), the light emitted by the sample was focused into an optical fiber and then guided to a CCD spectrometer synchronized with the current-voltage curve tracer. This allows us to measure the spectral emission of the device depending on the applied voltage or current. The electrical characterizations of the OFETs were performed by means of an Agilent E5273A 2 Channel (High Power, Medium Power) Source/Monitor Unit. The capacitance measurements were realized by using an impedance spectrometer Novocontrol Alpha-A High Performance Modular Measurement System. During the capacitance and OFET measurements, the samples were kept at room temperature and in N_2_ atmosphere.

Surface morphology characterization on the nanometer scale was performed by atomic force microscopy (AFM) using an Digital Instruments Dimension 3100 AFM operating in tapping mode. For quantitative surface roughness analysis, root-mean-square (rms) roughness *σ* and lateral correlation length *ξ* were calculated from 2.5 μm × 2.5 μm AFM image data by fitting the height-height correlation function: *C*(*x*)=*σ* ^2^exp[−(∣*x*∣/*ξ*)^2*α*^] [36].

## Results and discussion

### PSP/(PPNB:PVK) OLED

**Fig. 3:**
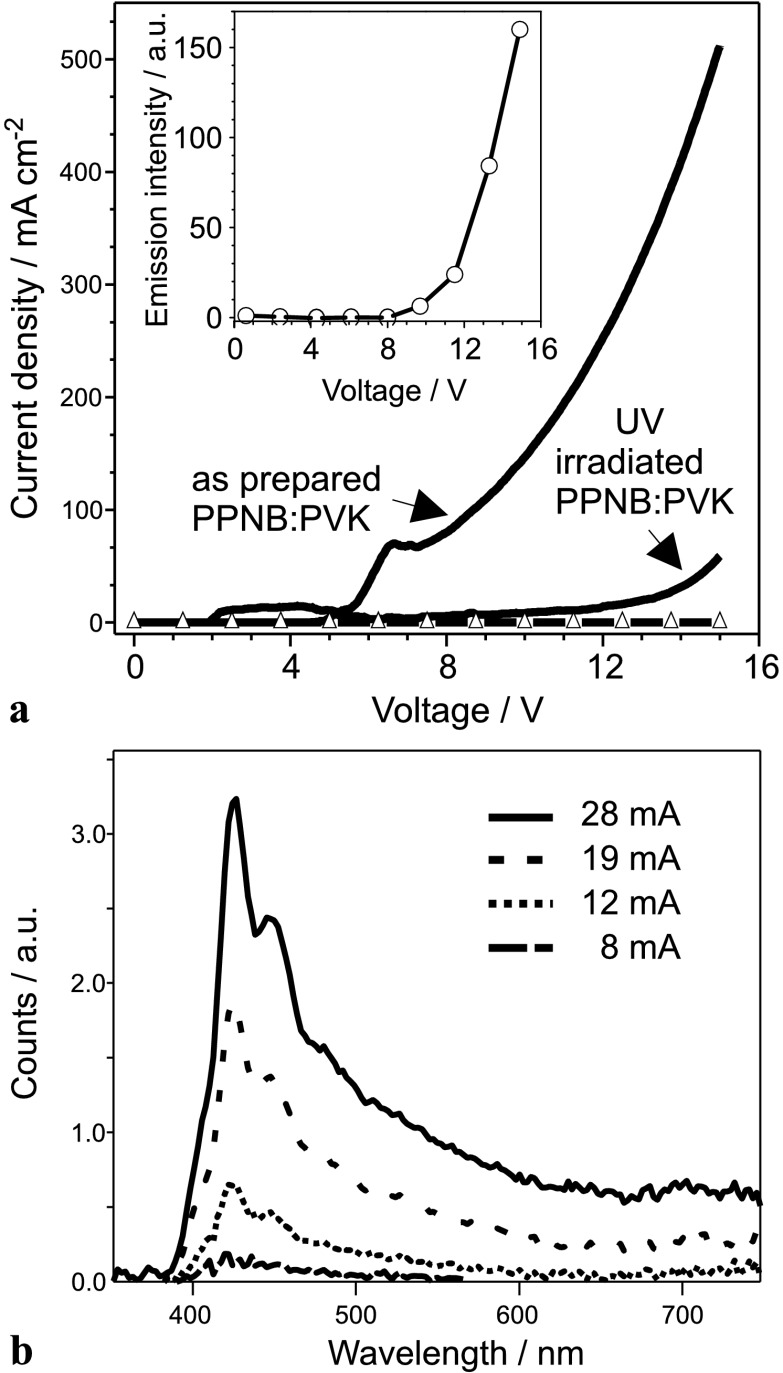
(**a**) The curve with triangles shows the I–V characteristics of an OLED built on pure PPNB. The continuous I–V curves are of devices fabricated with as prepared and UV-irradiated PPNB:PVK blends, respectively. The *insert* shows the electroluminescence emission as function of the applied voltage for the OLED built with as prepared PPNB:PVK blend. (**b**) Electroluminescence spectra versus driven current of OLED built with as prepared PPNB:PVK blend

The use of PPNB as UV photo sensitive material was tested in OLEDs. PPNB is a nonconductive polymer and, therefore, its inclusion as an interlayer in the structure of the OLED causes an increase of the threshold voltage. Hence, in order to increase the conductivity of the PPNB layer, it was mixed with PVK in a 1:1 mass ratio. The current–voltage (I–V) curves of OLEDs fabricated with interlayers of PPNB and PPNB:PVK are shown in Fig. 3(a), respectively. The addition of PVK to the PPNB increases the conductivity of the blend resulting in an OLED working with threshold voltage of just 5 V. The PPNB:PVK blend was then used as an interlayer in the OLED structure as shown schematically in Fig. 2(a).

The EL spectrum of an OLED fabricated on as prepared PPNB:PVK is shown in Fig. 3(b), where the intensity of the typical PSP emission increases as function of the driven current [27]. The emission spectrum is composed of several peaks with the maximum of the highest peak located at 425 nm. The spectra match the typical EL spectrum previously reported for PSP [27, 37], therefore, in our OLEDs, the PSP emission is not affected by the presence of the PPNB:PVK layer. Furthermore, the I–V characteristics of this OLED is presented in Fig. 3(a) showing a threshold voltage of approximately 5 V and a current flowing through the device close to 510 mA cm^−2^ for an applied voltage of 15 V. Consequently, it is demonstrated that the as prepared PPNB:PVK blend works as a conductive layer and its inclusion in OLEDs does not prevent the functionality of the device. The EL intensity of the device is plotted with open circles in the insert of Fig. 3(a) and it shows the expected increase in magnitude as the applied voltage rises up [27].

In contrast, the OLED fabricated with UV-illuminated PPNB:PVK does not show electroluminescence emission and the current flowing through it is significantly smaller than the current flowing through the non-UV-irradiated blend; see Fig. 3(a). Hence, for an applied voltage of 15 V, the current flowing through the UV-irradiated PPNB:PVK OLED is just 60 mA cm^−2^, while the current flowing through the OLED fabricated with the as-prepared blend reaches a value close to 510 mA cm^−2^. Simultaneously, the threshold voltage of the OLED with UV-illuminated PPNB:PVK is around 13 V, while for the OLED with as prepared PPNB:PVK film the threshold voltage is just 5 V.

**Fig. 4:**
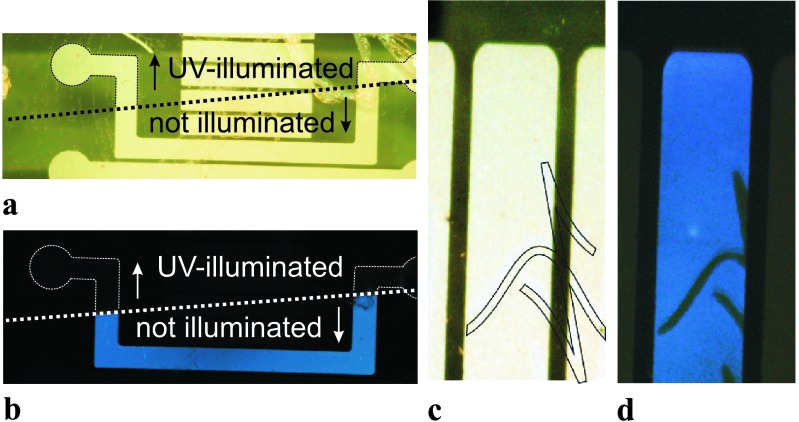
(**a**) Optical image of the sample after UV-irradiation. The dotted line indicates the border between the part of the sample which was UV irradiated and the other part which was covered by the shadow mask (as prepared PPNV:PVK). (**b**) Image of the blue light emitting PSP OLED in operation. (**c**) Images of the OLED patterned through a shadow mask with the logo of the Institute of Semiconductor and Solid State Physics, of the Johannes Kepler University, Linz. A drawing of the area UV-irradiated through the shadow mask, with the institute logo, is superimposed to the photo. (**d**) The device emitting blue light. The dark features in the centre correspond to areas of the UV-illuminated PPNB:PVK, where the EL is quenched. These features are around 100 μm wide

The obtained results show the possibility of patterning the light emission of PSP OLEDs by UV irradiation through shadow mask of the PPNB:PVK interlayer. Therefore, in order to test the proposed patterning method on a single device and to give an example of its practical application, a set of samples were prepared where one-half of the PPNB:PVK was kept in the dark, while the other half was UV-treated. An optical image of a sample is shown in Fig. 4(a). The areas corresponding to UV treated and as prepared PPNB are indicated. The part of the device built on the as prepared PPNB shows the typical PSP blue emission; see Fig. 4(b).

Furthermore, with the purpose of fabricating patterned OLEDs, a PSP OLED was fabricated on a PPNB:PVK blend UV patterned through a shadow mask containing the logo of the Institute of Semiconductor and Solid State Physics. Figure 4(c) presents an optical image of the device, the super imposed logo of the institute indicates the area of the sample treated with UV-light. Figure 4(d) shows the operating PSP OLED built using the patterned PPNB:PVK interlayer. A clear blue emission, characteristic for PSP electroluminescence [28, 37], is observed as well as areas with no emission. The 100 μm wide dark features, in the center of the emitting OLED, are part of the logo of the Institute and correspond to the area where the PPNB:PVK layer was UV-illuminated through the shadow mask.

**Fig. 5:**
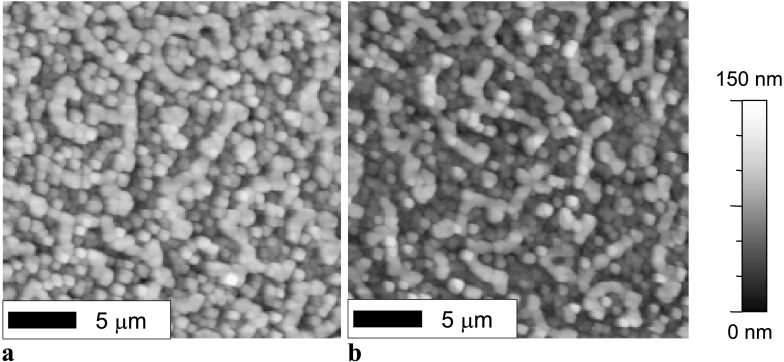
Surface morphology of PSP thermally evaporated on: (**a**) as prepared and (**b**) UV treated PPNB:PVK blend. The height scale is the same for both images

The AFM images of PSP layers on as-prepared and UV irradiated PPNB:PVK are presented in Figs. 5(a) and 5(b), respectively. The presented PSP layers were deposited at a substrate temperature of 100 °C for 60 minutes minutes. Both layers are formed by grains of ∼500 nm in size. The film deposited on the pristine PPNB:PVK presents an apparent higher density of crystallites than the one deposited on the UV treated blend. Nevertheless, such small difference in morphology cannot lead to the complete quenching of the PSP emission in the active layer deposited on the UV treated PPNB:PVK and, therefore, the explanation could be laying on the polymer blend photoreaction.

**Fig. 6:**
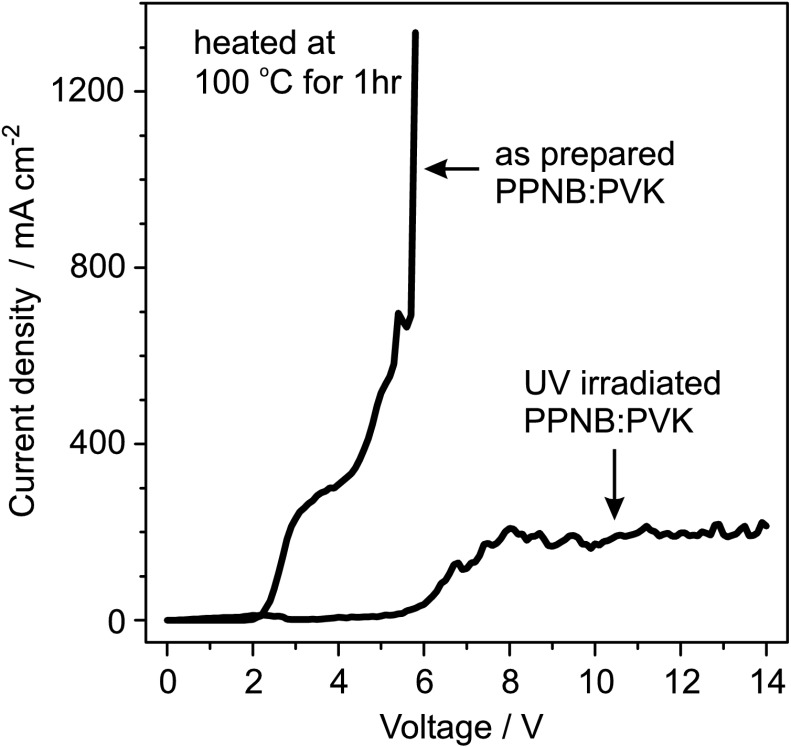
I–V characteristics of the PPNB:PVK blend alone

In order to clarify the influence of irradiation on the underlying layer of the PPNB:PVK blend, its conductivity was investigated by measuring the current voltage characteristic of sandwich structures glass/ITO/PEDOT:PSS/PPNB:PVK/Al, where each layer was prepared in the same manner as in the OLEDs. As shown in Fig. 6, the amount of current passing through the UV illuminated PPNB:PVK layer is smaller in comparison to the as prepared one. This means that the amount of charges injected for the same applied voltage is greatly reduced in the device fabricated with UV illuminated PPNB:PVK blend compared to the fabricated on the pristine polymer blend. The lower carrier injection is then found to be the source of the observed EL emission quenching and increased threshold voltage on the presented OLEDs; see Figs. 3(a) and 4(b). The most probable explanation for this decrease in conductivity could be found in the increased trap density caused by the photogenerated hydroxyl and ketone groups in the PPNB:PVK layer after the photo-Fries reaction.

### PPNB as gate dielectric in C_60_ OFETs

**Fig. 7:**
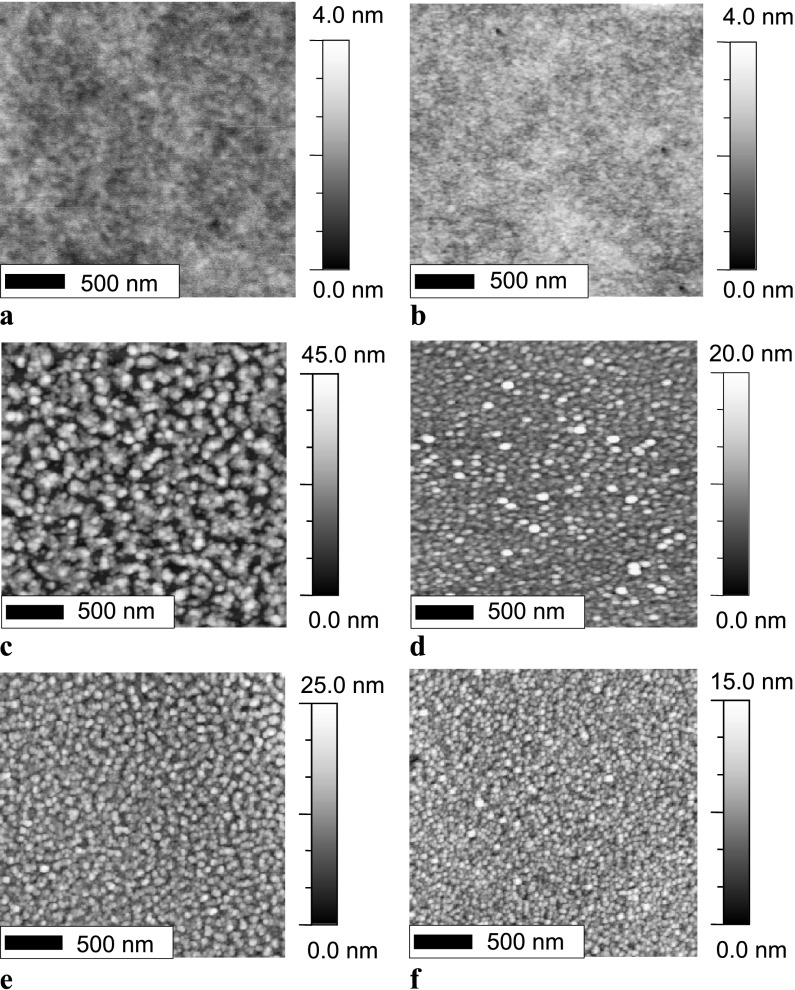
AFM images of bare PPNB substrates (**a**, **b**). AFM images of 10 nm (**c**, **d**) and 100 nm (**e**, **f**) thick C_60_ films, respectively, deposited on PPNB. The *left* and *right columns* are ascribed to nonilluminated and UV-illuminated PPNB, respectively

In OFETs, the transport of charge carriers takes place at the interface of the semiconductor with the gate dielectric [23–26]. Hence, the performance of such devices depends, to a large extent, on the physical properties of the gate dielectric, e.g., its dielectric constant, surface polarity, and density of charge traps [38–40]. In addition, the surface morphology of the gate dielectric and the morphology of the semiconductor deposited on it may influence the electrical properties of the OFETs [34, 41–43]. Therefore, we studied the influence of the UV illumination on the surface morphology of the thick PPNB film used as gate dielectric in our C_60_ OFETs and on the C_60_ films deposited on it. The surface morphology of the nonilluminated PPNB does not differ from that of the UV-illuminated one, as shown in the AFM Figs. 7(a) and 7(b), respectively. Both samples show very flat surfaces with an rms roughness of 0.3 nm. However, the surface morphology of the nominally 10 nm thick C_60_ is greatly influenced when thermally evaporated onto the UV-illuminated or as prepared PPNB layer. The growth of thermally evaporated C_60_ on nonilluminated PPNB is characterized by tall grains which are separated one from the other, similar to a column like growth, resulting in an rms roughness of 8.4 nm and lateral correlation length of 42 nm; see Fig. 7(c) [36]. In contrast, C_60_ deposited on UV-illuminated PPNB shows a film built up of smaller grains resulting in an rms roughness of 2.7 nm and a lateral correlation length of 35 nm, as shown in Fig. 7(d). Such growth difference is also observed for thicker C_60_ films. The morphology of a 100 nm thick C_60_ layer deposited on nonilluminated PPNB presents an rms roughness of 3.4 nm and a lateral correlation length of 25 nm; see Fig. 7(e). However, a C_60_ film of equal thickness deposited on UV-illuminated PPNB shows a smoother surface having an rms roughness of 2.1 nm and a lateral correlation length of 20 nm, as presented in Fig. 7(f).

**Fig. 8:**
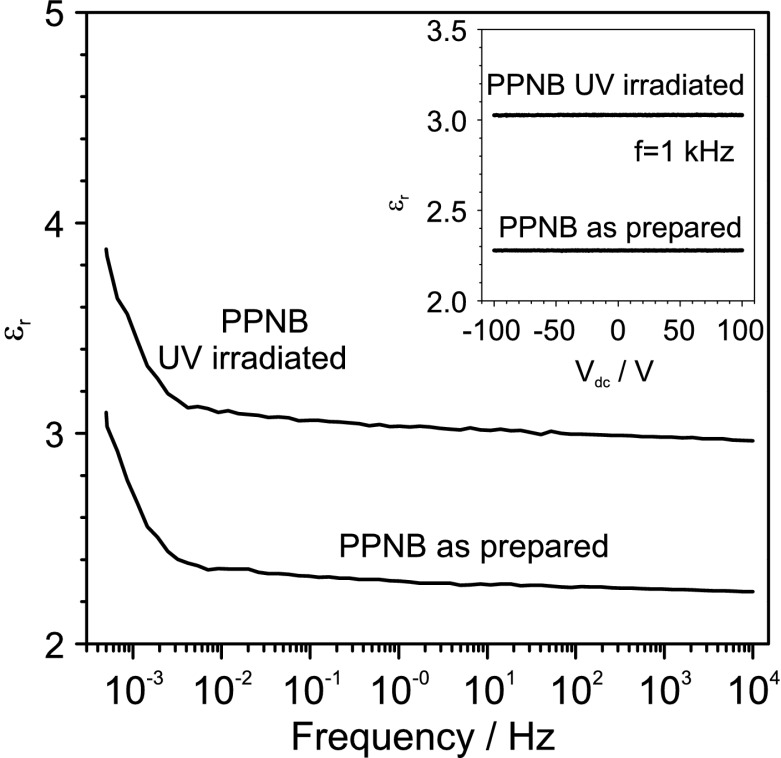
Frequency dependence of the dielectric constants of PPNB films, as prepared and UV-illuminated. The *insert* shows the dielectric constants of these films as function of the applied voltage. The measurement was carried out at a frequency of 1 kHz

The dielectric properties of 3.4 μm PPNB films were studied by means of capacitance measurements of MIM structures. Figure 8 shows that the UV-irradiated PPNB has a higher dielectric constant than the nonirradiated one. In addition, the dielectric constants of PPNB UV-illuminated and non-UV-illuminated, almost do not vary in the frequency range from 5×10^−2^ until 105 Hz, meaning that for a fixed gate voltage the amount of charges accumulated in the transistor channel are almost constant in the mentioned range of frequencies. Nevertheless, for very low frequencies, below 4×10^−2^ Hz, the dielectric constant increases in both cases. The insert of Fig. 8 shows the dielectric constants of these films as function of the applied voltage.

**Fig. 9:**
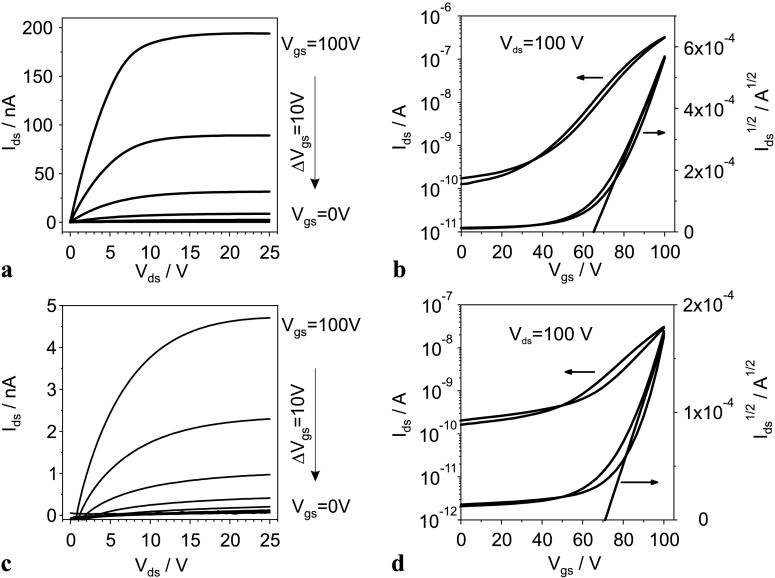
(**a**) and (**b**) Characteristics curves of C_60_ OFETs fabricated with as prepared PPNB. (**c**) and (**d**) Characteristics curves of C_60_ OFETs fabricated with UV-treated PPNB. The corresponding output curves are shown in plots (**a**) and (**c**). Semilogarithmic plot of *I*_ds_ vs. *V*_gs_ on the left axis of (**b**) and (**d**). Square root of *I*_ds_ vs. *V*_gs_ on the right axis of (**b**) and (**d**). The straight line corresponding to the linear fit to $I_{\mathrm{ds}}^{1/2}$ is also shown

The effect of UV illumination of the PPNB on the C_60_ OFETs performance was tested on devices built with the configuration shown in Fig. 2(b). The output characteristics of a device fabricated with as prepared PPNB gate dielectric is shown in Fig. 9(a), for a set of gate-source voltages (*V*_gs_). As observed the drain-source current (*I*_ds_), clearly saturates for drain-source voltages (*V*_ds_) higher than 10 V. Moreover, *I*_ds_ reaches its highest value of 1.9×10^−7^ A for *V*_gs_ equal to 100 V at *V*_ds_ as high as 25 V. Figure 9(b) shows in a semilogarithmic plot the dependence of *I*_ds_ as function of *V*_gs_ for *V*_ds_=100 V. The small hysteresis revealed by the *I*_ds_ curve proves the good performance and usability of the as prepared PPNB polymer as gate dielectric in OFETs.

As seen in the output curves presented in Fig. 9(c) the OFET prepared with UV-irradiated PPNB shows lower currents. In this case, the highest obtained value of *I*_ds_ is in the range of 10^−9^ A. The transfer curve of this device is presented in Fig. 9(d). This device also shows a small hysteresis.

It is worth to mention that for the studied OFETs no difference was found between the gate-source leakage currents (leakage current through the PPNB) (*I*_gs_) of the as prepared PPNB and the UV-treated PPNB. In all the cases, *I*_gs_ was in the range of 10^−10^ A for *V*_gs_ values around 50 V, while *I*_gs_ increases to the range of 2×10^−9^ A as *V*_gs_ reaches 100 V.

The field effect mobilities of the charge carriers and threshold voltages were calculated in the saturation regime by fitting the slope of the square root of *I*_ds_ vs. *V*_gs_ to a straight line; see the right axis of Figs. 9(b) and 9(d) [44]. The OFET built with nonirradiated PPNB has a field effect mobility of 3.2×10^−2^ cm^2^ V^−1^ s^−1^ and threshold voltages equal to 65 V for the fullerene based device. On the other hand, the OFET built with UV-irradiated PPNB has a field effect mobility one order of magnitude lower, 3.7×10^−3^ cm^2^ V^−1^ s^−1^ and the threshold voltages is 70 V.

Although the dielectric constant of PPNB increases upon UV-irradiation, the performance of the presented C_60_/PPNB transistors decreases contrarily to theory. However, there are two effects which can overrule the influence of the dielectric properties of the PPNB-layer. First, it is known that upon UV-irradiation the surface polarity of PPNB increases due to the formation of polar hydroxyl- and carbonyl groups [22]. These reactive groups directly at the transport channel of the OFET could contribute to an increase of charge trapping at the interface gate dielectric semiconductor [45]. In addition, as observed in the AFM images, the C_60_ shows smaller grain size when deposited on UV illuminated PPNB than when deposited on as prepared PPNB. It has been reported that the smaller the grain size the lower the performance of C_60_ OFETs [34, 43, 46]. A similar trend has also been observed for pentacene based OFETs [21, 47]. Consequently, both effects, will certainly lead to a lower charge carrier mobility and thus a decrease of the drain-source current, *I*_ds_. Therefore, it is possible to change the mobility of the C_60_, and thus the performance of the OFETs by the illumination time [38, 39].

## Conclusions

The effect of UV irradiation of PPNB:PVK and PPNB films on the growth of PSP and C_60_ films have been studied. PSP based OLEDs using a PPNB:PVK blend as interlayer were fabricated. The emission spectrum of the PSP OLED is not affected by the inclusion of the as prepared conductive PPNB:PVK film. However, the EL was quenched in those devices with PSP deposited on UV-illuminated interlayer. The performance of PPNB/C_60_ based OFETs is drastically reduced when the PPNB gate dielectric is UV-treated previously to the deposition of the C_60_ active layer. Such effects seem to be more correlated with an increase of the surface polarity of the PPNB and a smaller C_60_ grain size than with the observed increase of the dielectric constant of the PPNB upon UV-irradiation.

It has been demonstrated that the photosensitive polymer PPNB has potential use in the direct photolithographic patterning of devices based on evaporable organic semiconducting small molecules. The proposed method makes use of the direct patterning of the organic semiconducting layer by means of the photopatterning of the PPNB film without the need of removing or further treatment of the latter. Hence, it is a direct photopatterning process which does not require the chemical treatment of a photoresist in order to pattern the active layer of the electronic devices. The patterning, without optimization, of hundreds micrometer size features has been carried out. The photo-Fries reaction takes place only in UV illuminated areas of the PPNB or PPNB:PVK films. Therefore, the width of the delimiting border between as prepared areas and those areas where the photo-Fries reaction happens is not determined by the mechanism of the photo-Fries reaction itself. Indeed, in the proposed patterning method the resolution depends on the diffraction of light, namely on the applied wavelength and also on the thickness of the polymer layer. Thus, for the proposed method, a patterning resolution in the micrometer scale and even below is expected. The described method here could also be applied, for example, to Organic Read Only Memories where the working parameters of each diode or transistor is to be predefined during the fabrication process of the final product.
